# Human Gyrovirus in Healthy Blood Donors, France

**DOI:** 10.3201/eid1906.130228

**Published:** 2013-06

**Authors:** Philippe Biagini, Sandra Bédarida, Mhammed Touinssi, Vital Galicher, Philippe de Micco.

**Affiliations:** Unités Mixtes de Recherche Centre National de la Recherche Scientifique 7268 Equipe “Emergence et co-évolution virale,” Marseille, France

**Keywords:** gyrovirus, blood, human, France, blood donors, circovirus, viruses, GyV, human gyrovirus, HGyV, chicken anemia virus

**To the Editor:** Gyroviruses (GyVs) are naked, single-stranded DNA viruses that were described in chickens in 1979 (*1*). Chicken anemia virus (CAV), initially the sole member of the genus *Gyrovirus* (family *Circoviridae*), possesses a genome of ≈2.3 kb, containing 3 major partially overlapping open reading frames, viral proteins [VP] 1–3, and a short untranslated region (*1*). For >30 years, this virus, which was responsible for severe anemia and increased death rates in young chickens, was considered to have an extremely low genetic diversity and to be specific to this animal host. In 2011, however, sequence-independent molecular protocols enabled the characterization of highly divergent, GyV-related sequences in human and chicken biological samples. Human GyV 1 (HGyV1), avian GyV 2, and GyV3 sequences were identified from human skin, chicken blood, and human feces, respectively (*2*–*4*). These genomes harbor a genetic organization similar to CAV, despite a high genetic divergence (49%–65%).

One study described the detection of GyVs in HIV-positive patients and kidney transplant recipients (0.7% and 6%, respectively) (*5*), but these viruses had not been identified in blood samples from healthy persons. We investigated the presence of HGyV DNA in 352 blood samples from healthy blood donors in France (mean age 39 years; 185 men; M:F ratio 1:1.11). 

Plasma samples were prepared as described (*6*), and 1-mL aliquots were used for nucleic acids extraction (MagNA pure LC; Roche Diagnostics, Meylan, France). HGyV DNA was detected by using 2 systems in separate real-time TaqMan amplification assays (StepOne Plus; Applied Biosystems, Courtaboeuf, France). The first detection assay (VP1 gene) was described previously (HGyV-rtFP/HGyV-rtRP primers, HGyV-rtP probe, 72 nt) (*5*). The second assay was designed following the analysis of available HGyV sequences (Figure): sense primer HGyVsPBs 5′-GCTAAGACTGTRACATGGC-3′, reverse primer HGyVsPBr 5′-CTCCGGGAATAGCGTCTTC-3′, probe HGyVsPBp 5′-FAM-TGGCACTGGAGACACAGACTGCG-TAMRA-3′. This assay targets the VP2 gene of the viral genome, with an expected length of 118–115 bp, depending on the reference sequence considered. 

**Figure:**
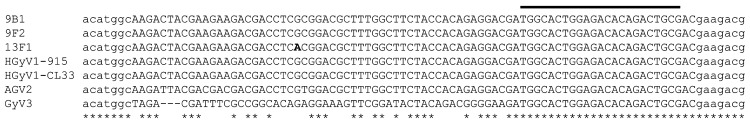
Alignment of partial sequences of human gyroviruses (HGyVs) from healthy blood donors, France. Point mutation corresponding to a nonsynonymous substitution is in **boldface** (13F1 isolate). Reference sequences and GenBank accession nos.: HGyV1-915, FR823283; HGyV1-CL33, JQ308212; avian gyrovirus (AGV) 2, JQ690763; gyrovirus (GyV) 3, JQ308210). Bar above sequences indicates location of the HGyVsPBp probe; lowercase letters indicate 5′/3′ ends of HGyVsPBs/HGyVsPBr real-time primers; asterisks (*) indicate conserved positions.

Amplification reactions were performed by using 10% of extracted material with the TaqMan Fast Universal PCR Kit (Applied Biosystems) in a final volume of 20 μL. Cycling conditions for both assays were 95°C for 20 s, followed by 50 cycles of 95°C for 1 s and 60°C for 20 s. The sensitivity of TaqMan assays was estimated to be 10 copies of HGyV DNA by using dilutions of a synthetic template. Each amplification product was subjected to additional agarose gel electrophoresis to help eliminate potential false-negative real-time PCR results.

Among the 352 plasma samples tested, 3 (0.85%) resulted in a positive signal by using our in-house real-time detection assay; no positive signal was identified by using the other system tested. When the tests were repeated, identical results were obtained. No additional amplicons were identifiable after agarose gel analysis. HGyV DNA titers in the 3 positive samples were low (<500 copies/mL plasma). Positive blood samples originated from 1 woman (age 44) and 2 men (ages 29 and 39). No biological or serologic marker evaluated for routine blood donor screening in France was associated with these donations. 

Partial HGyV sequences obtained were cloned and sequenced (7 clones each). All sequences characterized clustered with HGyV1 sequences, exhibiting either 100% nucleotide identity or 1 point mutation (corresponding to a nonsynonymous substitution, ala →thr) (Figure). No intragenetic diversity was identified.

Our results demonstrate that recently discovered HGyVs are detectable in blood of healthy persons. The low prevalence (0.85%) suggests, however, that the virus is infrequently found in the general population in France. A study from Italy of HGyVs in blood from healthy donors did not detect such viruses (*5*). This result may be linked to the small number of samples tested (n = 50) or to the use of a nonoptimized detection system designed on the basis of HGyV1 sequences only; moreover, the possibility that the VP1 and VP2 amplified regions would be conserved differently must be considered.

It is probable that subsequent sequences of HGyVs remain to be identified in human blood. A recent study reported the characterization of a highly divergent GyV sequence (GyV4) in human fecal samples and chicken meat (*7*); as with avian GyV2 and GyV3, further research is needed to determine whether this variant replicates in the human body or is solely ingested in food and passively excreted. A better knowledge of the genetic diversity of these newly discovered viruses will enable development of improved molecular detection systems and their subsequent use in epidemiologic studies involving diverse human cohorts.

The potential clinical importance of HGyVs remains to be clarified. Although infection with CAV in birds is frequently associated with clinical signs and disease, the presence of HGyVs in immunocompromised or immunocompetent humans does not appear to be correlated with visible symptoms. Further studies of the natural history and distribution of HGyVs in human hosts are needed.
